# Sequence-Specific Inhibition of Small RNA Function

**DOI:** 10.1371/journal.pbio.0020098

**Published:** 2004-02-24

**Authors:** György Hutvágner, Martin J Simard, Craig C Mello, Phillip D Zamore

**Affiliations:** 1Department of Biochemistry and Molecular Pharmacology, University of Massachusetts Medical SchoolWorcester, MassachusettsUnited States of America; **2**Program in Molecular Medicine, University of Massachusetts Medical SchoolWorcester, MassachusettsUnited States of America; **3**Howard Hughes Medical Institute, University of Massachusetts Medical SchoolWorcester, MassachusettsUnited States of America

## Abstract

Hundreds of microRNAs (miRNAs) and endogenous small interfering RNAs (siRNAs) have been identified from both plants and animals, yet little is known about their biochemical modes of action or biological functions. Here we report that 2′*-O-*methyl oligonucleotides can act as irreversible, stoichiometric inhibitors of small RNA function. We show that a 2′*-O-*methyl oligonucleotide complementary to an siRNA can block mRNA cleavage in *Drosophila* embryo lysates and HeLa cell S100 extracts and in cultured human HeLa cells. In Caenorhabditis elegans, injection of the 2′-*O*-methyl oligonucleotide complementary to the miRNA *let-7* can induce a *let-7* loss-of-function phenocopy. Using an immobilized 2′*-O-*methyl oligonucleotide, we show that the C. elegans Argonaute proteins ALG-1 and ALG-2, which were previously implicated in *let-7* function through genetic studies, are constituents of a *let-7*-containing protein–RNA complex. Thus, we demonstrate that 2′*-O-*methyl RNA oligonucleotides can provide an efficient and straightforward way to block small RNA function in vivo and furthermore can be used to identify small RNA-associated proteins that mediate RNA silencing pathways.

## Abstract 2

2′-*O*-methyl RNA oligonucleotides can block small RNA function in vivo - providing a tool to determine the biological functions of siRNA and microRNA. These oligonucleotides can also be used to identify small RNA-associated proteins that mediate silencing pathways

## Introduction

The endoribonuclease Dicer produces two types of small regulatory RNAs that regulate gene expression: small interfering RNAs (siRNAs) and microRNAs (miRNAs) (Bernstein et al. 2001; Grishok et al. 2001; Hutvágner et al. 2001; Ketting et al. 2001; Knight and Bass 2001). In animals, siRNAs direct target mRNA cleavage (Elbashir et al. 2001b, 2001c), whereas miRNAs block target mRNA translation (Lee et al. 1993; Reinhart et al. 2000; Brennecke et al. 2003; Xu et al. 2003). Recent data suggest that both siRNAs and miRNAs incorporate into similar, perhaps even identical, protein complexes and that a critical determinant of mRNA destruction versus translation regulation is the degree of sequence complementary between the small RNA and its mRNA target (Hutvágner and Zamore 2002; Mourelatos et al. 2002; Zeng et al. 2002; Doench et al. 2003; Saxena et al. 2003; Zeng et al. 2003).

Target RNA cleavage directed by siRNA is called RNA interference (RNAi). RNAi is a powerful method for the study of gene function in animals and plants and has even been proposed as a therapy for treating genetic disorders and viral infections. Biochemical studies in *Drosophila* S2 cells (Bernstein et al. 2001; Hammond et al. 2001a; Caudy et al. 2002; Liu et al. 2003) and affinity purification (Martinez et al. 2002) or immunoprecipitation (Hutvágner and Zamore 2002) from cultured human HeLa cells have identified protein components of the RNAi effector complex, the RNA-induced silencing complex (RISC). Genetic mutations that disrupt RNAi in Caenorhabditis elegans, *Drosophila*, green algae, fungi, and plants have likewise identified proteins required for RNAi (Cogoni and Macino 1997, 1999a, 1999b; Ketting et al. 1999; Tabara et al. 1999, 2002; Catalanotto et al. 2000, 2002; Dalmay et al. 2000, 2001; Fagard et al. 2000; Grishok et al. 2000; Ketting and Plasterk 2000; Mourrain et al. 2000; Wu-Scharf et al. 2000; Grishok and Mello 2002; Tijsterman et al. 2002a, 2002b). Key steps in the RNAi pathway have also emerged from studies of RNAi reconstituted in cell-free extracts (Tuschl et al. 1999; Zamore et al. 2000; Hammond et al. 2001b; Nykänen et al. 2001; Martinez et al. 2002; Schwarz et al. 2002; Tang et al. 2003).

Recently, hundreds of miRNAs have been identified in animals and plants (Lagos-Quintana et al. 2001, 2002; Lau et al. 2001; Lee and Ambros 2001; Reinhart et al. 2002; Ambros et al. 2003; Aravin et al. 2003; Brennecke and Cohen 2003; Lim et al. 2003). Of these, the biological functions of only four animal miRNAs are known. In C. elegans, the miRNAs *lin-4* (Lee et al. 1993; Olsen and Ambros 1999) and *let-7* (Reinhart et al. 2000) regulate developmental timing, whereas the *Drosophila* miRNAs *bantam* and *miR-14* control cell survival by repressing translation of proapoptotic genes (Brennecke et al. 2003; Xu et al. 2003). Computational approaches promise to identify the mRNA targets of other miRNAs (Enright et al. 2003; Lewis et al. 2003; Stark et al. 2003), but these proposed miRNA/target mRNA pairs will require experimental confirmation. Despite the widespread use of RNAi to “knock down” gene function, the RNAi pathway itself remains poorly understood. Similarly, despite intensive efforts to identify all miRNAs in vertebrates, worms, and flies, the mechanisms underlying miRNA function remain mysterious and no biological function has been ascribed to the vast majority of miRNAs.

Here we show that 2′-*O*-methyl oligonucleotides are potent and irreversible inhibitors of small RNA-directed RNA silencing in vivo and in vitro. Our experiments using 2′-*O*-methyl oligonucleotides also demonstrate that the acquisition of a target RNA by an siRNA-programmed RISC is far more efficient than the binding of an antisense oligonucleotide to the same region of the target. To demonstrate the utility of 2′-*O*-methyl oligonucleotides in probing RNA silencing pathways, we show that 2′*-O-*methyl oligonucleotides efficiently block siRNA-directed RISC activity in cell extracts and in cultured human HeLa cells. When injected into C. elegans larvae, a *let-7*-complementary 2′-*O*-methyl oligonucleotide can efficiently suppress *lin-41* translational repression by the *let-7* miRNA. Finally, we use a tethered 2′-*O*-methyl oligonucleotide to demonstrate association of the C. elegans Argonaute proteins ALG-1 and ALG-2 with *let-7*.

## Results

### Inhibition of RNAi by 2′-*O*-Methyl Oligonucleotides

Although RNAi has proved a straightforward and cost-effective method to assess the function of protein-coding mRNAs (Fire et al. 1998; Caplen et al. 2000, 2001; Carthew 2001; Elbashir et al. 2001a) and even some noncoding RNAs (Liang et al. 2003), no comparable method allows the sequence-specific inactivation of the siRNA or miRNA components of the RISC. The ideal inhibitor of RISC function would be a nucleic acid that would be recognized by the RISC by nucleotide complementarity, but be refractory to RISC-directed endonucleolytic cleavage or translational control. In theory, such a molecule would titrate out RISCs containing a complementary siRNA or miRNA, but not affect the function of RISCs containing guide RNAs unrelated in sequence. Such a RISC inhibitor should also resist degradation by cellular ribonucleases so that it persists long enough to bind RISC and block its function. Finally, the ideal inhibitor of small RNA function would act at concentrations unlikely to elicit nonspecific responses to the inhibitor itself, i.e., in the low nanomolar range, the same concentration at which siRNAs themselves are effective.

**Figure 1:**
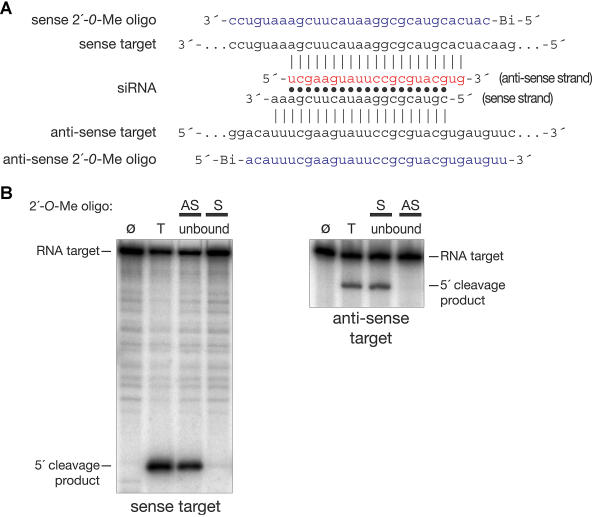
A 2′*-O-*Methyl RNA Oligonucleotide Inhibits RNAi In Vitro in *Drosophila* Embryo Lysate (A) Sequences of the sense and antisense *Pp*-luc target RNAs (black), the siRNA (red, antisense strand; black, sense strand), and the sense and antisense 2′*-O-*methyl oligonucleotides (blue) used. (B) Sequence-specific depletion of RNAi activity by immobilized 2′-*O*-methyl oligonucleotides from *Drosophila* embryo lysate programmed with siRNA. siRNA was incubated with lysate to assemble RISC; then, immobilized 2′-*O*-methyl oligonucleotide was added. Finally, the beads were removed from the supernatant, and either sense or antisense ^32^P-radiolabeled target RNA was added to the supernatant to measure RISC activity for each siRNA strand. Symbols and abbreviations: Ø, target RNA before incubation with siRNA-programmed lysate; T, total reaction before depletion; unbound, the supernatant after incubation with the immobilized antisense (AS) or sense (S) 2′-*O*-methyl oligonucleotides shown in (A). The absence of 5′ cleavage product demonstrates that the sense oligonucleotide depleted RISC containing antisense siRNA, but not sense siRNA, and the antisense oligonucleotide depleted the sense RISC, but not that containing antisense siRNA. Bi, 5′ biotin attached via a six-carbon linker.

At micromolar concentration, DNA antisense oligonucleotides may block miRNA function in *Drosophila* embryos (Boutla et al. 2003), but the poor stability of DNA oligonucleotides in vivo may limit their utility. Phosphorothioate-substituted DNA oligonucleotides, which show good in vivo stability, do not inhibit RISC function in vitro (data not shown). 2′-*O*-methyl oligonucleotides are also highly resistant to cellular ribonucleases (Inoue et al. 1987). To test whether 2′-*O*-methyl oligonucleotides can act as RISC inhibitors, we asked whether a 2′-*O*-methyl oligonucleotide, tethered to streptavidin paramagnetic beads via a 5′ biotin linkage, could be used to deplete siRNA-programmed RISC from the reaction. *Drosophila* embryo lysate was programmed with a synthetic siRNA duplex directed against a firefly (Photinus pyralis) luciferase (*Pp*-luc) mRNA target (Figure 1A). Then, a tethered 31-nt 2′-*O*-methyl oligonucleotide complementary to the 21-nt siRNA antisense strand was added. Finally, the beads were removed from the solution using a magnet, and the supernatant was tested for siRNA-programmed RISC activity. Under these conditions, the 2′-*O*-methyl oligonucleotide completely depleted the reaction of the RISC programmed with the antisense strand of the siRNA, but not of RISC programmed with the sense strand (Figure 1B). Thus, depletion occurred only when the siRNA strand contained within RISC was complementary to the tethered oligonucleotide.

**Figure 2:**
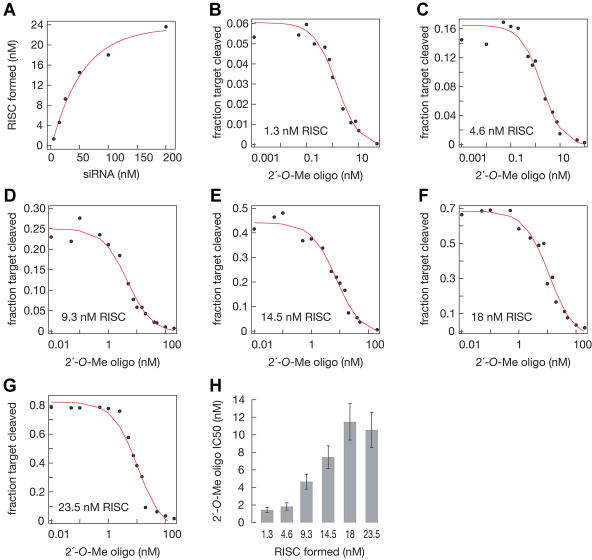
2′-*O*-Methyl Oligonucleotides Act as Stoichiometric, Irreversible Inhibitors of RISC Function (A) The immobilized sense 2′-*O*-methyl oligonucleotide was used to determine the concentration of ^32^P-radiolabeled antisense siRNA assembled into RISC in *Drosophila* embryo. The 2′-*O*-methyl oligonucleotide and siRNA duplex are shown in Figure 1A. (B–G) Inhibition of RNAi was measured using free 2′-*O*-methyl oligonucleotide and 1.3 nM (B), 4.6 nM (C), 9.3 nM (D), 14.5 nM (E), 18 nM (F), and 23.5 nM (G) RISC. The concentration of 2′-*O*-methyl oligonucleotide required for half-maximal inhibition (IC_50_) was calculated by fitting each dataset to a sigmoidal curve using a Hill coefficient of 1. (H) A plot of IC_50_ versus RISC concentration suggests that each 2′-*O*-methyl oligonucleotide binds a single RISC. The data suggest that binding is essentially irreversible.

We extended this method to measure the amount of RISC formed in the in vitro reaction at different concentrations of the siRNA duplex. An siRNA duplex in which the antisense strand was 5′-^32^P-radiolabeled was incubated in the reaction; then, the tethered 2′-*O*-methyl oligonucleotide was added to deplete the reaction of antisense siRNA-programmed RISC. The beads were then washed and the fraction of ^32^P-siRNA bound to the beads determined. Depletion was verified by testing the supernatant for RISC activity. Formally, the amount of ^32^P-siRNA retained on the beads for a given concentration of siRNA duplex places an upper limit on the concentration of RISC formed. However, our results using this assay are, within error, identical to the amount of RISC measured by two independent methods: the accumulation of single-stranded siRNA from functionally asymmetric siRNA duplexes (Schwarz et al. 2003), and the magnitude of the burst of target cleavage measured by pre-steady-state kinetics (B. Haley and P. D. Zamore, unpublished data). The simplest explanation for our results is that this assay directly measures siRNA incorporated into RISC. Figure 2A shows the results of this assay for six different concentrations of siRNA duplex (5 nM, 15 nM, 25 nM, 50 nM, 100 nM, and 200 nM siRNA). First, the data show that RISC assembly in vitro is inefficient; the majority of siRNA duplexes do not contribute to RISC production. Second, RISC assembly is saturable, suggesting that some component of RISC itself is limiting.

To understand better the mechanism by which the 2′-*O*-methyl oligonucleotide interacted with RISC, we measured the concentration of free 2′-*O*-methyl oligonucleotide required for half-maximal inhibition of RISC activity (IC_50_; Figure 2B–2G) at the six different RISC concentrations determined in Figure 2A. The IC_50_ for inhibition by free 2′-*O*-methyl oligonucleotide is shown for each RISC concentration in Figure 2H. The IC_50_ for the 2′-*O*-methyl oligonucleotide was remarkably close to half the RISC concentration, suggesting that a single 31-nt 2′-*O*-methyl oligonucleotide binds each RISC and blocks its function. Consistent with this apparent 1:1 stoichiometry, the data for the 2′-*O*-methyl oligonucleotide titrations fit well to sigmoidal curves, with a Hill coefficient of 1 (Figure 2B–2G). The sequence specificity of 2′-*O*-methyl oligonucleotide inhibition of RISC function clearly shows that inhibition reflects binding of the oligo to the RISC. Our data are most easily explained if the concentration of the 2′-*O*-methyl oligonucleotide required for inhibition in these experiments is much greater than the K_D_ for binding; i.e., the experiments were conducted in a stoichiometric binding regime. Under a stoichiometric binding regime, inhibition by the 2′-*O*-methyl oligonucleotides would be essentially irreversible.

**Figure 3:**
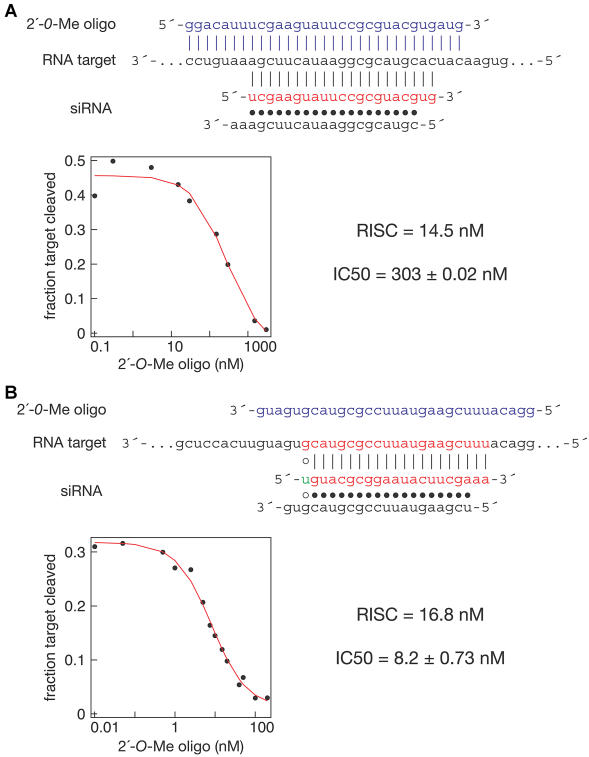
RISC Does Not Act through an Antisense Mechanism (A) Inhibition of sense target cleavage by an antisense 2′*-O-*methyl oligonucleotide requires an approximately 40-fold higher concentration than by a sense oligonucleotide. The antisense oligonucleotide can pair completely with the sense target RNA, but not with the antisense siRNA-programmed RISC. The IC_50_ value and the RISC concentration are indicated. Also shown are the sequences of the sense *Pp*-luc RNA target (black), the siRNA (red, antisense strand; black, sense strand), and the 2′*-O-*methyl oligonucleotide (blue). (B) The same antisense 2′-*O*-methyl oligonucleotide is an effective competitor of antisense target cleavage. In this experiment, inhibition occurs via binding of the antisense oligonucleotide to the sense siRNA-programmed RISC, not the target RNA. The IC_50_ value and the RISC concentration are indicated. Also shown are the sequences of the *Pp*-luc antisense RNA target (black), the siRNA (red, antisense strand; black, sense strand), and the 2′*-O-*methyl oligonucleotide (blue). The G:U wobble in the siRNA duplex in (B) acts to direct the sense strand into RISC and improving its efficacy in target cleavage.

In theory, the 2′*-O*-methyl oligonucleotide might act by displacing the passenger (sense) strand of the siRNA duplex, thereby blocking incorporation of the guide (antisense) strand into RISC (Elbashir et al. 2001c). We can exclude this possibility because a 5′ tethered 31-nt 2′-*O*-methyl oligonucleotide complementary to the passenger strand of the siRNA did not deplete the guide-strand RISC activity (see Figure 1B). Similarly, an antisense sequence 2′-*O*-methyl oligonucleotide cannot pair with antisense RISC, but can pair with a sense target mRNA. We anticipated that this antisense 2′-*O*-methyl oligonucleotide would pair with the sense target mRNA and occlude the antisense RISC from the target. Surprisingly, the antisense 2′-*O*-methyl oligonucleotide was a poor inhibitor of antisense RISC function when it was used to bind the target site, requiring 300 nM for half-maximal inhibition in a reaction containing 14.5 nM RISC and 3 nM sense target RNA (Figure 3A). By contrast, the same antisense 2′-*O*-methyl oligonucleotide was highly effective in blocking the activity of the sense RISC, to which it is complementary, acting with an IC_50_ of 8.2 nM in a reaction containing 16.8 nM sense-strand RISC and 3 nM antisense target RNA (Figure 3B). (In this experiment, sense-strand RISC was generated by changing the first nucleotide of the sense strand from C to U, thereby reversing its functional asymmetry [Schwarz et al. 2003].)

Thus, the interaction of 2′-*O*-methyl oligonucleotide with RISC is dramatically different from the interaction of 2′-*O*-methyl oligonucleotide with target RNA; RISC has a more than 40-fold greater affinity for the 2′-*O*-methyl oligonucleotide than the oligonucleotide has for an RNA target (compare Figures 2E and 3A). These data imply that the interaction of RISC with target is more complex than simple nucleic acid hybridization. Inhibition of the siRNA-programmed RISC by a 2′-*O*-methyl oligonucleotide with the sequence of the target RNA is more effective than inhibition mediated by binding of an oligonucleotide to the target RNA itself. Thus, the RISC is more adept at finding or remaining bound (or both) to the target RNA than a 2′-*O*-methyl oligonucleotide. These data suggest that specific proteins in the RISC facilitate either target finding, target binding, or both. Consistent with this idea, inhibition of RISC function was incomplete using 21-nt 2′-*O*-methyl oligonucleotides (data not shown). Thus, target sequence flanking the site of complementarity to the siRNA guide strand may play a role in target RISC binding. Perhaps an active mechanism that involves target sequences flanking the siRNA facilitates the search for the target sequence; future studies will clearly be needed to test this idea.

### Inhibition of RNAi in Cultured Human Cells

**Figure 4:**
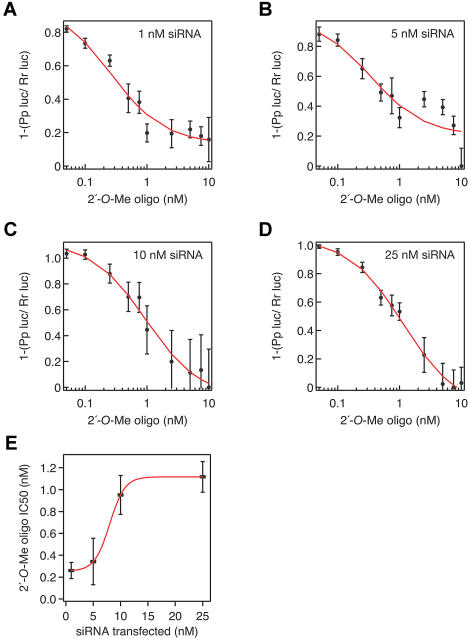
A 2′-*O*-Methyl Oligonucleotide Is a Potent Inhibitor of RNAi in Human Cultured HeLa Cells (A–D) HeLa cells were transfected with 1 nM (A), 5 nM (B), 10 nM (C), or 25 nM (D) siRNA-targeting *Pp-*luc mRNA. The next day the cells were cotransfected with *Rr-*luc*-*and *Pp*-luc-expressing plasmids together with various amounts of a 31-nt 2′*-O-*methyl oligonucleotide complementary to the antisense strand of the siRNA. The half-maximal concentration of 2′-*O*-methyl oligonucleotide required to inhibit (IC_50_) was determined by fitting the data to a sigmoidal curve using a Hill coefficient of 1. (E) IC_50_ plotted as a function of the concentration of transfected siRNA.

Our data show that 2′*-O-*methyl oligonucleotides are stoichiometric, irreversible, sequence-specific inhibitors of siRNA function in RNAi reactions using *Drosophila* embryo lysate. Can 2′-*O*-methyl oligonucleotides block siRNA function in vivo? To address this question, we carried out sequential transfection experiments using 1 nM, 5 nM, 10 nM, or 25 nM siRNA duplex. siRNA was transfected on the first day; then, reporter and control plasmids were cotransfected together with various amounts of 2′-*O*-methyl oligonucleotide on the second day. Silencing of *Pp*-luc, relative to the sea pansy (Renilla reniformis) luciferase (*Rr*-luc) control, was measured on the third day. For each siRNA concentration, we determined the concentration of 2′-*O*-methyl required for half-maximal inhibition of RNAi (Figure 4A–4D). Increasing amounts of the 2′-*O*-methyl oligonucleotide gradually extinguished the ability of the siRNA to silence *Pp*-luc in all four experiments. The inhibition of silencing in the cultured cells cannot be a consequence of the 2′-*O*-methyl oligonucleotide displacing the sense strand of the siRNA duplex, because assembly of siRNA into RISC occurred a full day before the oligonucleotide was introduced. When 10 nM siRNA was used in the transfection, approximately 1 nM 2′-*O*-methyl RNA was required for half-maximal inhibition of RNAi (Figure 4C and 4E). At 25 nM siRNA, approximately 1.1 nM 2′-*O*-methyl RNA was required to inhibit half the RNAi activity (Figure 4D and 4E). In Figure 4E, we plot the siRNA concentration versus the amount of 2′-*O*-methyl oligonucleotide required for half-maximal inhibition of silencing (IC_50_). The data fit well to a sigmoidal curve, consistent with the idea that, at these concentrations, increasing amounts of siRNA do not produce a corresponding increase in RISC activity. Higher concentrations of siRNA were not examined because they produce sequence-independent changes in gene expression (Persengiev et al. 2003; Semizarov et al. 2003). We conclude that both cells and extracts have a limited capacity to assemble RISC on exogenous siRNA. Our data suggest that the use of siRNA concentrations greater than that required to produce the maximum amount of RISC will lead to the accumulation of double-stranded siRNA in vivo and may thus contribute to the undesirable, sequence-independent responses sometimes observed in cultured mammalian cells (Persengiev et al. 2003; Semizarov et al. 2003; Sledz et al. 2003).

### Inhibition of miRNA Function In Vitro and In Vivo

**Figure 5:**
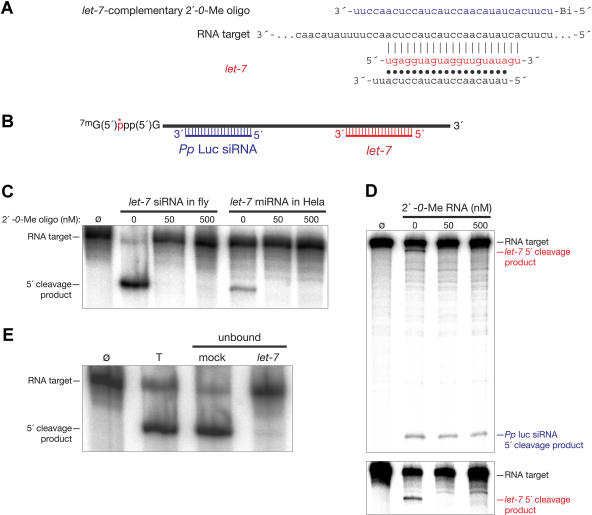
A Complementary 2′*-O-*Methyl Oligonucleotide Blocks Endogenous *let-7*-Containing RISC Function (A) Sequence of the *let-7*-complementary site in the target RNA (black), of the siRNA (red, antisense strand; black, sense strand), and of the *let-7*-complementary 2′*-O-*methyl oligonucleotide (blue). (B) Schematic representation of the target RNA, which contained both *Pp*-luc and antisense *let-7* sequences. (C) *Drosophila* embryo lysate (left) was programmed with *let-7* siRNA; then, the target RNA and the 2′*-O-*methyl oligonucleotide were added together. Target RNA and 2′-*O*-methyl oligonucleotide (right) were added to HeLa S100 extract, which contains endogenous human *let-7*-programmed RISC. (D) An RNA target containing both *Pp*-luc and antisense *let-7* sequence can be simultaneously targeted by *Pp*-luc siRNA and endogenous *let-7* in HeLa S100 lysate. The *let-7*-complementary 2′-*O*-methyl oligonucleotide blocks *let-7*-programmed, but not *Pp*-luc siRNA-programmed, RISC function. The bottom panel shows the same samples analyzed separately to better resolve the *let-7* 5′ cleavage product. (E) *Drosophila* embryo lysate was programmed with *let-7* siRNA and then incubated with biotinylated 2′-*O*-methyl oligonucleotide tethered to paramagnetic streptavidin beads. The beads were removed and the supernatant tested for RNAi activity. Symbols and abbreviations: Ø, target RNA before incubation with siRNA-programmed lysate; T, total reaction before depletion; unbound, the supernatant after incubation with the paramagnetic beads. “Mock” indicates that no oligonucleotide was used on the beads; “*let-7*” indicates that the beads contained the *let-7*-complementary oligonucleotide shown in (A).

In animal cells, miRNAs are thought predominantly to function as translational regulators. Nonetheless, a growing body of evidence suggests that they function through a similar, if not identical, RISC as siRNAs (Hutvágner and Zamore 2002; Zeng et al. 2002, 2003; Doench et al. 2003; Khvorova et al. 2003; Schwarz et al. 2003). Because 2′-*O*-methyl oligonucleotides block siRNA function in vitro and cultured human cells, we asked whether these oligonucleotides might likewise disrupt the function of a specific miRNA in vitro and in vivo. An ideal candidate for such an miRNA is *let-7*. Classical genetic mutations in *C. elegans let-7* produce well-characterized, readily scored phenotypes. Furthermore, human HeLa cells express multiple *let-7* family members, and endogenous *let-7* is present naturally in RISC (Hutvágner and Zamore 2002; Zeng and Cullen 2003). We tested whether a 31-nt 2′*-O-*methyl oligonucleotide complementary to *let-7* could block target cleavage guided by the endogenous *let-7*-programmed RISC present in HeLa S100 extract (Figure 5A). (Our assay detects the target-cleaving activity of *let-7*; we have not examined endogenous human mRNA targets whose translation may be repressed by *let-7*.) As a control, we also tested whether the oligonucleotide could block the activity of a *let-7*-containing RISC assembled in vitro in *Drosophila* embryo lysate. Addition of this 2′-*O*-methyl oligonucleotide efficiently blocked target RNA cleavage directed by the endogenous *let-7*-programmed RISC in the HeLa S100 extract and by the RISC programmed with exogenous *let-7* siRNA duplex in *Drosophila* embryo lysate (Figure 5C).

In addition to containing endogenous *let-7*-programmed RISC, HeLa S100 can be programmed with exogenous siRNA duplexes (Martinez et al. 2002; Schwarz et al. 2002). The target RNA used in Figure 5B also contains sequence from the *Pp*-luc mRNA and can therefore be targeted by a *Pp*-luc-specific siRNA duplex (see Figures 1A and 5C). We incubated the *Pp*-luc siRNA duplex with the human HeLa S100 to form *Pp*-luc-directed RISC, then added the *let-7*-complementary 2′-*O*-methyl oligonucleotide and the target RNA. The oligonucleotide blocked cleavage by the endogenous *let-7*-programmed RISC, but had no effect on cleavage directed by the exogenous *Pp*-luc siRNA in the same reaction (Figure 5D). When tethered to a paramagnetic bead, this oligonucleotide could also quantitatively deplete the *let-7*-programmed RISC from the *Drosophila* embryo lysate (Figure 5E), demonstrating that, again, the interaction between the 2′-*O*-methyl oligonucleotide and the RISC was apparently irreversible. The 2′-*O*-methyl oligonucleotide was a specific and potent inhibitor of target cleavage directed by a naturally occurring, miRNA-programmed RISC. Furthermore, our data demonstrate that individual RISCs act independently even when they target the same RNA.

**Figure 6:**
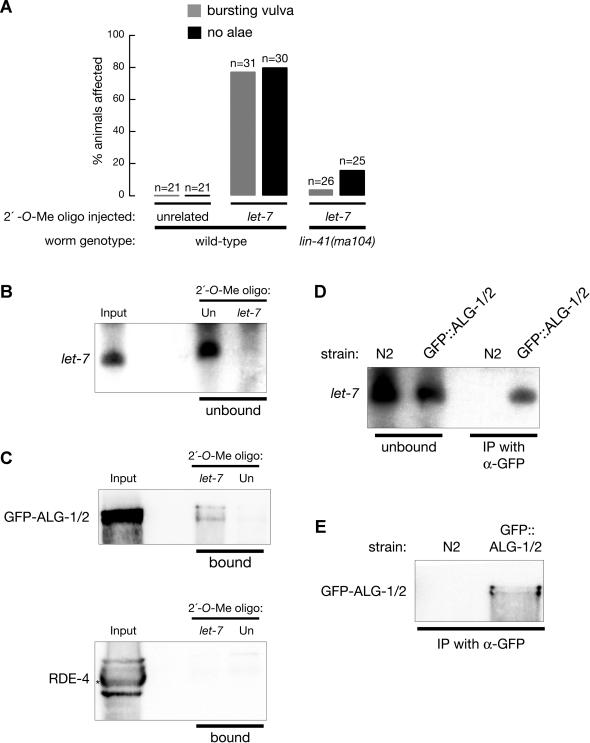
Injection of a 2′-*O*-Methyl Oligonucleotide Complementary to *let-7* miRNA Can Phenocopy the Loss of *let-7* Function in C. elegans (A) Wild-type and *lin-41(ma104)* L2-stage C. elegans larvae were injected with either a 2′*-O-*methyl oligonucleotide complementary to *let-7* miRNA (Figure 5A) or an unrelated *Pp*-luc 2′-*O*-methyl oligonucleotide. Absence of alae and presence of bursting vulvae were scored when the injected animals reached adulthood. (B) Isolation of *let-7*-associated proteins with a tethered 2′-*O*-methyl oligonucleotide. Northern blot analysis of *let-7* miRNA remaining in the supernatant of the worm lysate after incubation with the *let-7*-complementary (*let-7*) or *Pp*-luc (unrelated) oligonucleotide. Input represents the equivalent of 50% of the total extract incubated with tethered oligonucleotide. (C) Western blot analysis of the GFP-tagged ALG-1 and ALG-2 proteins associated with *let-7*. The upper band corresponds to GFP-tagged ALG-1 and the lower to GFP-tagged ALG-2. Extracts from a transgenic strain expressing the tagged proteins was incubated with the indicated tethered 2′-*O*-methyl oligonucleotide; then, the beads were washed and bound proteins were fractionated on an 8% SDS-polyacrylamide gel. Western blots were probed using anti-GFP monoclonal or anti-RDE-4 polyclonal antibody. The RDE-4-specific band is marked with an asterisk (Tabara et al. 2002). (D and E) Analysis of *let-7* miRNA in ALG-1/ALG-2 complexes (D). Extracts prepared from mixed-stage wild-type worms (N2) or from GFP::ALG-1/ALG-2 transgenic worms were immunoprecipitated using anti-GFP monoclonal antibodies. The unbound and immunoprecipitated RNAs were analyzed by Northern blot hybridization for *let-7* (D), and 5% of the immunoprecipitated protein was analyzed by Western blotting for GFP to confirm recovery of the GFP-tagged ALG-1/ALG-2 proteins (E).

Next we asked whether 2′-*O*-methyl oligonucleotides can inhibit miRNA function in vivo. Translational repression directed by miRNAs occurs in C. elegans, where both the *lin-4* and *let-7* miRNAs have been shown to block translation of their target mRNAs without altering mRNA stability (Wightman et al. 1993; Ha et al. 1996; Moss et al. 1997; Olsen and Ambros 1999; Reinhart et al. 2000; Seggerson et al. 2002). The genetics of *lin-4* and *let-7* function are well-characterized in worms, where they are required during larval development to control the timing and pattern of cell division in the hypodermis (Lee et al. 1993; Reinhart et al. 2000). First, we tested whether injection into the germline of wild-type adult hermaphrodites of 2′*-O-*methyl oligonucleotides complementary to either *lin-4* or *let-7* could block *lin-4* or *let-7* function during the larval development of the resulting progeny. Although the 2′*-O-*methyl oligonucleotides were not toxic and when coinjected with an unrelated DNA transformation reporter did not prevent the uptake and expression of the coinjected DNA, we did not observe inhibition of *lin-4* or *let-7* activity (data not shown). This finding suggests that single-stranded 2′*-O-*methyl oligonucleotides are not efficiently transmitted to the progeny of injected animals. To circumvent this problem, we next injected 2′*-O-*methyl oligonucleotides directly into larvae and examined the phenotypes of the injected animals. The *lin-4* miRNA functions in L1/L2 larvae, and we have found that, in our hands, L1 larvae do not survive microinjection (data not shown); thus, it was not possible to assay for inhibition of *lin-4* function by direct injection. In contrast, *let-7* functions during the L4 stage, and we found that L2 and L3 larvae survive the microinjection procedure (see Materials and Methods). Loss of *let-7* function causes worms to reiterate the L4 larval molt and inappropriately produce larval cuticle at the adult stage. Loss-of-function *let-7* phenotypes include weak cuticles prone to bursting at the vulva, defects in egg-laying, and loss of adult-specific cuticular structures that run the length of the animal's body, the alae (Reinhart et al. 2000). After larvae were injected with the *let-7*-specific 2′*-O-*methyl oligonucleotide, 80% of the adult worms lacked alae; 77% lacked alae and also exhibited bursting vulvae (Figure 6A). In contrast, animals injected with an unrelated control 2′*-O-*methyl oligonucleotide displayed no abnormal phenotypes (Figure 6A).

All of the phenotypes associated with injection of the *let-7*-complementary 2′*-O-*methyl oligonucleotide are consistent with a loss of *let-7* activity. *let-7* represses translation of *lin-41* mRNA by binding to a partially complementary site in the *lin-41* 3′-untranslated region (Reinhart et al. 2000; Slack et al. 2000; Vella et al. 2004). Consequently, many of the phenotypes associated with the loss of *let-7* reflect overexpression of LIN-41 protein; *let-7* mutants are partially suppressed by mutations in *lin-41*. We reasoned that if the phenotypes observed in the injected larvae reflect a loss of *let-7* activity, then they should likely be partially suppressed by a *lin-41* mutation (Reinhart et al. 2000; Slack et al. 2000). To test this possibility, we injected the *let-7*-specific 2′-*O*-methyl oligonucleotide into the *lin-41(ma104)* strain and compared the penetrance of the phenotypes with those observed for injection into wild-type*.* Consistent with the idea that the injected oligonucleotide specifically inactivates *let-7*, the absence of alae- and vulval-bursting phenotypes were both suppressed in the *lin-41(ma104)* mutant strain (Figure 6A). The number of worms lacking alae was reduced from 80% to 16%, and worms with bursting vulvae were dramatically reduced (74% in wild-type compared to 3.8% in the *lin-41(ma104)* strain). The observed suppression (64%) was nearly identical to that reported for a *let-7, lin-41* genetic double mutant (70%; Reinhart et al. 2000; Slack et al. 2000). Together, our data support the idea that 2′*-O-*methyl oligonucleotides are potent inhibitors of miRNA function that can be used to probe the function of specific miRNAs in C. elegans.

### Isolation of Protein–miRNA Complex Using a Tethered 2′-O-Methyl Oligonucleotide

Our in vitro experiments suggest that both siRNA- and miRNA-containing RISCs are stably bound by 2′*-O-*methyl oligonucleotides. In theory, tethered 2′-*O*-methyl oligonucleotides could be used to isolate cellular factors associated with specific miRNAs. In human cells, miRNAs such as *let-7* are in a protein complex that contains Argonaute proteins (Hutvágner and Zamore 2002; Mourelatos et al. 2002; Dostie et al. 2003). In C. elegans, the Argonaute protein-encoding genes *alg-1* and *alg-2* are required for the biogenesis or function (or both) of the miRNAs *lin-4* and *let-7* (Grishok et al. 2001), but it has not been shown whether ALG-1 and ALG-2 proteins are directly associated with *let-7*.

We prepared extracts from wild-type adult worms carrying a transgene expressing GFP-tagged ALG-1 and ALG-2 proteins. The extracts were then incubated with the *let-7*-complementary 2′*-O-*methyl oligonucleotide tethered by a 5′ biotin to streptavidin-conjugated paramagnetic beads. As a control, the experiment was performed in parallel using an oligonucleotide not complementary to *let-7*. The *let-7*-complementary, but not the control, oligonucleotide depleted nearly all the *let-7* miRNA from the extract (Figure 6B). Western blotting using an anti-GFP antibody revealed that both GFP-tagged ALG-1 and ALG-2 protein copurified with the *let-7*-complementary oligonucleotide, but not the control oligonucleotide (Figure 6C). In contrast, the RNA-binding protein RDE-4, which is required for RNAi but not for miRNA function in C. elegans, did not copurified with the *let-7*-complementarity oligonucleotide, providing further support for the specificity of the *let-7*:ALG-1/ALG-2 interaction (Figure 6C).

Finally, we used a coimmunoprecipitation assay to examine the interaction between *let-7* and ALG-1/ALG-2. In this assay, a monoclonal anti-GFP antibody was used to coimmunoprecipitate ALG-1/ALG-2 and small RNAs from the GFP::ALG-1/GFP::ALG-2 strain, which expresses GFP::ALG-1/ALG-2 fusion proteins. Northern blot analysis of the immune complex showed that it contained mature 22-nt *let-7* miRNA (Figure 6D). No detectable *let-7* was recovered with the anti-GFP antibody from the N2 wild-type strain. By comparing the fraction of *let-7* associated with GFP::ALG-1/ALG-2 with the unbound fraction of *let-7* miRNA, we estimate that approximately 30% of the 22-nt *let-7* RNAs coimmunoprecipitate with GFP::ALG-1 and GFP::ALG-2. These data support a model in which that ALG-1 and ALG-2 form a complex, in vivo*,* that contains a substantial fraction of the mature *let-7* miRNA.

## Discussion

Our studies indicate that 2′-*O*-methyl oligonucleotides bind efficiently and essentially irreversibly to RISC by basepairing with the small guide RNA. These findings provide a rapid and reliable method to measure programmed RISC concentration in vitro and to identify the in vivo functions of small RNA and the identities of their associated proteins. The ability to measure RISC concentration should enable detailed kinetic studies of the enzymatic activity of RISC, an essential step in understanding RISC function. In fact, this method was recently put to use in analyzing the molecular basis of asymmetry in siRNA function (Schwarz et al. 2003). In this study, we have used a tethered 2′-*O*-methyl oligonucleotide to demonstrated the association of ALG-1/ALG-2, two C. elegans Argonaute proteins, with the endogenous worm miRNA *let-7*.

Our in vitro and in vivo studies using 2′-*O*-methyl oligonucleotides demonstrate that cells and extracts have a limited capacity to assemble RISC on exogenous siRNA. Our in vitro experiments suggest that inhibition of RISC by 2′-*O*-methyl oligonucleotides is stoichiometric and essentially irreversible. Using a sequential transfection protocol in cultured cells, we find that the half-maximal amount of 2′-*O*-methyl oligonucleotide required to inhibit silencing (IC_50_) is less than the amount of siRNA transfected. These data suggest that only a fraction of the transfected siRNA forms RISC. Furthermore, the data are consistent with stoichiometric and irreversible binding of the 2′-*O*-methyl oligonucleotide to RISC in vivo.

Our data hint that recognition of the 2′-*O*-methyl oligonucleotide by RISC and, by inference, recognition of target RNA by RISC are qualitatively different from the simple binding of two complementary nucleic acids by basepairing. We observed that RISC function was far more readily inhibited by binding a 2′-*O*-methyl oligonucleotide to RISC than by binding the same 2′-*O*-methyl oligonucleotide to the site of RISC recognition on a target RNA. A clear implication of this finding is that RISC does not acquire its RNA target by a passive basepairing mechanism that zippers together 21 nt of complementary RNA. Thus, RNAi is not merely a form of antisense inhibition in which the antisense strand is stabilized in a duplex. Rather, an active mechanism—perhaps involving target sequences flanking the region of complementarity—underlies the specificity and efficiency of RISC targeting.

Finally, we have shown the utility of 2′*-O-*methyl oligonucleotides to probe miRNA function in vivo. Injection of a 2′-*O*-methyl oligonucleotide complementary to the *let-7* miRNA into C. elegans larvae phenocopied a *let-7* loss-of-function mutation, demonstrating that 2′*-O-*methyl oligonucleotides can disrupt the function of a single miRNA in vivo. These data, combined with our studies in vitro and in cultured cells, show the promise of 2′-*O*-methyl oligonucleotides as a tool for dissecting the function of the numerous miRNAs found in a wide range of organisms. In this regard, 2′-*O*-methyl oligonucleotides provide a tool similar in practice, but mechanistically distinct from, RNAi itself and thus may facilitate the study of small RNA function in cases in which classical genetic mutations in miRNA genes are unavailable.

## Materials and Methods

### 

#### General methods

*Drosophila* embryo lysate preparation, in vitro RNAi reactions, and cap-labeling of target RNAs were as described elsewhere (Haley et al. 2003). Target RNAs were used at approximately 3 nM concentration. Cleavage products of RNAi reactions were analyzed by electrophoresis on 5% or 8% denaturing polyacrylamide gels. Gels were dried, exposed to image plates, and then scanned with a FLA-5000 phosphorimager (Fuji Photo Film Company, Tokyo, Japan). Images were analyzed using Image Reader FLA-5000 version 1.0 (Fuji) and Image Gauge version 3.45 (Fuji). Data analysis was performed using Excel (Microsoft, Redmond, Washington, United States) and IgorPro 5.0 (Wavemetrics, Lake Oswego, Oregon, United States).

#### siRNA and 2′-*O*-methyl oligonucleotides

Synthetic siRNA (Dharmacon, Lafayette, Colorado, United States) was deprotected according to the manufacturer, annealed (Elbashir et al. 2001b, 2001c), and used at 50 nM final concentration unless otherwise noted. 2′-*O*-methyl oligonucleotides (either from IDT, Santa Clara, California, United States, or from Dharmacon) were 5′-CAU CAC GUA CGC GGA AUA CUU CGA AAU GUC C-3′ and 5′-Bio-CAU CAC GUA CGC GGA AUA CUU CGA AAU GUC C-3′ (complementary to the *Pp*-luc siRNA sense strand); 5′-GGA CAU UUC GAA GUA UUC CGC GUA CGU GAU G-3′ and 5′-Bio-A CAU UUC GAA GUA UUC CGC GUA CGU GAU GUU-3′ (complementary to the *Pp*-luc antisense strand); and 5′-Bio-UCU UCA CUA UAC AAC CUA CUA CCU CAA CCU U-3′ (complementary to *let-7*); 5′ biotin was attached via a six-carbon spacer arm.

#### Immobilized 2′-*O*-methyl oligonucleotide capture of RISC

Biotinylated 2′-*O*-methyl oligonucleotide (10 pmol) was incubated for 1 h on ice in lysis buffer containing 2 mM DTT with 50 μl of Dynabeads M280 (as a suspension as provided by the manufacturer; Dynal, Oslo, Norway) to immobilize the oligonucleotide on the beads. To ensure that the tethered oligonucleotide remained in excess when more than 50 nM siRNA was used, 20 pmol of biotinylated 2′-*O*-methyl oligonucleotide was immobilized. For RISC capture assays, siRNA was preincubated in a standard 50 μl in vitro RNAi reaction for 15 min at 25°C. Then, the immobilized 2′-*O*-methyl oligonucleotide was added to the reaction and incubation continued for 1 h at 25°C. After incubation, beads were collected using a magnetic stand (Dynal). The unbound supernatant was recovered and an aliquot assayed for RISC activity as previously described (Elbashir et al. 2001b; Nykänen et al. 2001) to confirm that RISC depletion was complete. The beads were then washed three times with ice-cold lysis buffer containing 0.1% (w/v) NP-40 and 2 mM DTT, followed by a wash without NP-40. To determine the amount of RISC formed, input and bound radioactivity was determined by scintillation counting (Beckman Instruments, Fullerton, California, United States). To isolate *let-7*-containing complexes from C. elegans adults, we incubated 20 pmol of immobilized 2′-*O*-methyl oligonucleotide with 1 mg of total protein.

#### Sequential transfection

HeLa S3 cells were transfected in a 24-well plate (200 mm^2^ per well) using Lipofectamine 2000 (GIBCO, San Diego, California, United States) according to the manufacturer's protocol, first with various concentrations of siRNA targeting *Pp-*luc mRNA. After 6 h, the cells were washed with PBS and the media replaced. On the next day, the cells were cotransfected with *Rr*-luc-expressing (0.1 μg/well) and *Pp*-luc-expressing (0.25 μg/well) plasmids and 2′-*O*-methyl oligonucleotides using Lipofectamine 2000 (GIBCO) according to the manufacturer's protocol. The luciferase activity was measured 24 h later with the Dual Luciferase assay kit (Promega, Madison, Wisconsin, United States) using a Mediators Diagnostika (Vienna, Austria) PhL luminometer.

#### Worm injection

For in vivo inhibition of *let-7* function, 1 mg/ml *let-7*-complementary 2′-*O*-methyl oligonucleotide in water (100 μM) was injected into either wild-type (N2) or *lin-41(ma104)* L2 larvae. Injection of L2 larvae was essentially as described elsewhere (Conte and Mello 2003). The 2′-*O*-methyl oligonucleotide solution was injected into the body cavity of the larvae using the low flow and pressure setting to prevent animals from dying. Despite these precautions, approximately 60% of the animals do not survive injection, irrespective of the oligonucleotide injected. *let-7* phenotypes were also observed at 10 μM oligonucleotide, but were less penetrant. Phenotypes were scored after the injected animals survived to adulthood.

#### Other methods

Synchronized transgenic animals carrying GFP::ALG-1, GFP::ALG-2 were harvested at adulthood and homog-enized in ice-cold buffer (25 mM HEPES–NaOH [pH 7.4], 150 mM NaCl, 1 mM EDTA, 1 mM DTT, 10% [v/v] glycerol, 0.5% [v/v] Triton X-100, 2% [v/v] SUPERaseIn [Ambion, Austin, Texas, United States]) and Mini Complete Protease Inhibitor cocktail (1 tablet/10 ml solution) (Roche, Basel, Switzerland) using a stainless-steel Dounce homogenizer (Wheaton Incorporated, Millville, New Jersey, United States). The homogenized extract was clarified by a centrifugation at 13,817 × *g* for 10 min at 4°C.

To recover the proteins associated with the *let-7* miRNA, the beads were boiled for 10 min in 20 μl of SDS loading buffer (10 mM Tris–HCl [pH 6.8], 2% [w/v] SDS, 100 mM DTT, and 10% [v/v] glycerol). Proteins were resolved by SDS-PAGE on an 8% gel and transferred to Hybond-C membrane (Amersham Biosciences, Little Chalfont, United Kingdom). To detect GFP-tagged ALG-1, ALG-2, and RDE-4 proteins, the membrane was incubated overnight at 4°C with either monoclonal anti-GFP (Roche) or an affinity-purified polyclonal anti-RDE-4 antibody (Tabara et al. 2002) diluted 1:1000 into TBST-milk solution (100 mM Tris–HCl [pH 7.5], 150 mM NaCl, 0.1% [v/v] Tween-20, and 5% [w/v] dried milk), incubated 1 h at room temperature with either anti-mouse (GFP-tagged ALG-1/ALG-2) or anti-rabbit (RDE-4) HRP-conjugated secondary antibody (Jackson Laboratory, Bar Harbor, Maine, United States) diluted 1:5,000 in TBST and then visualized by enhanced chemulinescence (New England Nuclear, Boston, Massachusetts, United States).

Immunoprecipitation of GFP-tagged ALG-1/ALG-2 complexes was performed by preclearing worm extract with 50 μl of protein G–agarose beads (Roche) per 5 mg of total protein for 1 h at 4°C. The cleared extract was then incubated with 10 μg of monoclonal antibody anti-AFP 3E6 (Qbiogene, Montreal, Quebec, Canada) for 1 h at 4°C followed by 50 μl of protein G–agarose. The agarose beads were then washed three times with ice-cold homogenization buffer.

Depletion of *let-7* miRNA was monitored by Northern blotting. RNA was eluted from the immobilized 2′-*O*-methyl oligonucleotide by digestion with 1 mg/ml proteinase K in 200 mM Tris–HCl (pH 7.5), 25 mM EDTA, 300 mM NaCl, 2% (w/v) SDS at 50°C for 30 min, followed by extraction with phenol–chloroform, and recovered by precipitation with ethanol. Recovered RNA was resuspended in 10 μl of formamide-loading buffer (98% [v/v] deionized formamide, 10 mM EDTA, 0.025% [w/v] xylene cyanol, 0.025 % [w/v] bromophenol blue), heated to 100°C for 2 min. RNA was resolved on a 15% denaturing polyacrylamide gel, transferred to Hybond-N membrane (Amersham Biosciences), and detected by Northern blot analysis using a 5′-^32^P-radiolabeled antisense *let-7* RNA probe (UAU ACA ACC UAC UAC CUC AUU) as described elsewhere (Hutvágner and Zamore 2002).

## Supporting Information

### Accession Numbers

The GenBank (http://www.ncbi.nlm.nih.gov/Genbank/) accession number for Photinus pyralis is X65324 and for Renilla reniformis is AF025846.

Rfam (http://www.sanger.ac.uk/Software/Rfam/index.shtml) accession numbers for the *let-7* family members are MI0000060–MI0000068, MI0000433, and MI0000434.

The LocusLink (http://www.ncbi.nlm.nih.gov/LocusLink/) ID numbers for the genes discussed in this paper are *alg-1* (181504), *alg-2* (173468), *bantam* (117376), *let-7* (266954), *lin-4* (266860), *lin-41* (172760), *miR-14* (170868), and *rde-4* (176438).

## Abbreviations

miRNA: microRNA
*Pp*-luc: Photinus pyralis (firefly) luciferase
RISC: RNA-induced silencing complex
RNAi: RNA interference
*Rr*-luc: Renilla reniformis (sea pansy) luciferase
siRNA: small interfering RNA
